# Clinically-relevant consecutive treatment with isoproterenol and adenosine protects the failing heart against ischaemia and reperfusion

**DOI:** 10.1186/1479-5876-12-139

**Published:** 2014-05-21

**Authors:** Igor Khaliulin, Andrew P Halestrap, Simon M Bryant, Declan J Dudley, Andrew F James, M-Saadeh Suleiman

**Affiliations:** 1 School of Clinical Sciences and Bristol Cardiovascular, University of Bristol, Bristol Royal Infirmary, Upper Maudlin Street, Bristol BS2 8HW, UK

**Keywords:** Ischaemia/reperfusion, Cardioprotection, Isoproterenol, Adenosine, Heart failure, Mitochondria permeability transition pore

## Abstract

**Background:**

Consecutive treatment of normal heart with a high dose of isoproterenol and adenosine (Iso/Ade treatment), confers strong protection against ischaemia/reperfusion injury. In preparation for translation of this cardioprotective strategy into clinical practice during heart surgery, we further optimised conditions for this intervention using a clinically-relevant dose of Iso and determined its cardioprotective efficacy in hearts isolated from a model of surgically-induced heart failure.

**Methods:**

Isolated Langendorff-perfused rat hearts were treated sequentially with 5 nM Iso and 30 μM Ade followed by different durations of washout prior to 30 min global ischaemia and 2 hrs reperfusion. Reperfusion injury was assessed by measuring haemodynamic function, lactate dehydrogenase (LDH) release and infarct size. Protein kinase C (PKC) activity and glycogen content were measured in hearts after the treatment. In a separate group of hearts, Cyclosporine A (CsA), a mitochondria permeability transition pore (MPTP) inhibitor, was added with Iso/Ade. Failing hearts extracted after 16 weeks of ligation of left coronary artery in 2 months old rats were also subjected to Iso/Ade treatment followed by ischaemia/reperfusion.

**Results:**

Recovery of the rate pressure product (RPP) in Iso/Ade-treated hearts was significantly higher than in controls. Thus in Iso/Ade treated hearts with 5 nM Iso and no washout period, RPP recovery was 76.3 ± 6.9% of initial value vs. 28.5 ± 5.2% in controls. This was associated with a 3 fold reduction in LDH release irrespective to the duration of the washout period. Hearts with no washout of the drugs (Ade) had least infarct size, highest PKC activity and also showed reduced glycogen content. Cardioprotection with CsA was not additive to the effect of Iso/Ade treatment. Iso/Ade treatment conferred significant protection to failing hearts. Thus, RPP recovery in failing hearts subjected to the treatment was 69.0 ± 16.3% while in Control hearts 19.7 ± 4.0%. LDH release in these hearts was also 3 fold lower compared to Control.

**Conclusions:**

Consecutive Iso/Ade treatment of normal heart can be effective at clinically-relevant doses and this effect appears to be mediated by glycogen depletion and inhibition of MPTP. This intervention protects clinically relevant failing heart model making it a promising candidate for clinical use.

## Background

Reperfusion following a prolonged period of ischaemia induces myocardial dysfunction and necrotic damage [1]. Ischaemia and reperfusion–elicited tissue injury contributes to morbidity and mortality in a wide range of pathologies and is a major challenge during organ transplantation and cardiothoracic, vascular and general surgery [2].

We have previously described a cardioprotective protocol termed temperature preconditioning (TP) [3] where a few brief episodes of hypothermic perfusion (at 26°C) interspersed with normothermic perfusion (at 37°C) significantly improved heart resistance to the subsequent index ischaemia. The cardioprotective effect of TP, which exceeds the effect of ischaemic preconditioning, is associated with activation of protein kinases A (PKA) and C (PKC) and can be abolished by inhibitors of PKA and PKC (H-89 and chelerythrine, respectively) [3,4]. Furthermore, a combination of TP with the PKA inhibitor H-89 (which has no direct effect on PKC) reduced activation of PKC indicating that in the mechanism of TP, PKC activation occurs downstream to activation of PKA [4]. Importantly, TP mediated by the consecutive activation of PKA and PKC protects hearts during global normothermic ischaemia and during hypothermic ischaemia and cardioplegia [5]. When the consecutive activation of PKA and PKC was mimicked pharmacologically using the PKA activator isoproterenol (Iso) and the PKC activator adenosine (Ade), it resulted in a highly potent cardioprotective effect [4]. This was achieved using a concentration of Iso (200 nM) significantly higher than the concentration of isoproterenol in blood plasma used clinically (0.02-0.2 μg/kg/min; equivalent to about 1.3-13 nM) [6]. In contrast, the concentration of adenosine used in our experiments (30 μM) is considerably lower than that used for blood cardioplegia during cardiac surgery (1–2 mM) [7,8]. Therefore it is important to test both drugs at concentrations that are clinically relevant. Furthermore, in our earlier work investigating this intervention, we had not explored what duration of the washout period prior to ischaemia produced the maximal cardioprotective effect by triggering signalling mechanisms.

It is now recognized that opening of the mitochondria permeability transition pore (MPTP) plays a key role in mediating the irreversible damage of the myocardium during reperfusion [9] and that a range of interventions that inhibit pore opening, either directly or indirectly, are cardioprotective [10-12]. This was confirmed for the cardioprotective effect of consecutive Iso/Ade treatment with Iso used at relatively high concentration [4] but has not been investigated at a clinically relevant concentration. This can be achieved by establishing whether the cardioprotective effects of Iso/Ade treatment are additive to those of cyclosporine A (CsA) which is one of the most effective inhibitors of MPTP [13] widely used in experiments on isolated hearts.

The presence of co-morbid illness can render the myocardium resistant to cardioprotection against infarction by physical or pharmacological stimuli [14]. Thus an important and necessary step towards translation of this cardioprotective strategy into clinical practice is to study the efficacy of consecutive Iso/Ade treatment in diseased hearts.

Here we study whether our treatment is effective at low, clinically relevant concentrations of Iso and optimize the protocol of the consecutive Iso/Ade treatment. We confirm the underlying mechanism to be inhibition of MPTP opening and that the treatment is effective in a surgically-induced model of heart failure.

## Methods

Unless otherwise stated, all biochemicals were purchased from Sigma and general chemicals from Fischer Scientific or VWR-Jencons.

### Experimental Procedures

#### Heart perfusion and analysis of haemodynamic function

All procedures conform to the Directive 2010/63/EU of the European Parliament and the Guide for the Care and Use of Laboratory Animals published by the US National Institutes of Health (NIH Publication No. 85–23, revised 1996). Ethical approval was granted by the University of Bristol, UK. Two months old male Wistar rats (250–260 g) were killed by stunning and cervical dislocation. Hearts (~0.75 g) were rapidly removed into ice-cold Krebs-Henseleit buffer (KH) containing (mm): NaCl 118, NaHCO_3_ 25, KCl 4.8, KH_2_PO_4_ 1.2, MgSO_4_ 1.2, glucose 11 and CaCl_2_ 1.2, gassed with 95% O_2_-5% CO_2_ at 37 °C (pH 7.4) and perfused in Langendorff mode. The aorta was rapidly cannulated and the heart perfused at a rate of 8 ml · min^−1^ · g^−1^ heart weight with in-line filter using KH. Monitoring of left ventricular pressure was performed with a water-filled balloon inserted into the left ventricle, set to give an initial left ventricular end diastolic pressure (LVEDP) of 2.5–5 mmHg. All hearts were allowed an equilibration period of at least 25 min before any additional treatments was given. Data acquisition and analysis used a PowerLab System (ADInstruments, Australia). Left ventricular developed pressure (LVDP) was calculated as the difference between left ventricular systolic pressure (LVSP) and LVEDP, and work index (RPP) as the product of LVDP and heart rate (HR). Time derivatives of pressure were measured during contraction (+dP/dt) and relaxation (−dP/dt). The haemodynamic parameters were computed using the software Chart 5 (ADInstruments, Australia). Preischaemic values of the parameters of haemodynamic function were measured at the end of the equilibration period prior to any preischaemic intervention. Since the hearts in our experiments contracted spontaneously, haemodynamic function is reflected in both LVDP and HR. For this reason, RPP was considered as the main parameter characterising haemodynamic function. If RPP was less than 15,000 mmHg·beat/min at the end of the preischaemic equilibration period, the heart was excluded from the experiment.

#### Model of Surgically-Induced Heart Failure

Heart failure was surgically induced by ligation of the left coronary artery as described previously [15] and modified in our laboratory. In brief, 2 month old rats were anesthetized with ketamine (75 mg/kg, i.p.) and medetomidine (0.5 mg/kg, i.p.). Analgesia was induced by buprenorphine (0.05 mg/kg, s.c.). The rats were intubated and ventilated with a respirator for small animals (Harvard Apparatus). Body temperature was maintained at 37°C during the surgical procedure. An incision was made in the left side of the chest to expose the fourth intercostal space. A 7–0 surgical suture was placed under the left coronary artery and tied to complete occlusion of the artery. In sham-operated animals (SO), the coronary artery was not ligated. The wound was closed with a 4–0 surgical suture. Atipamezole (2.5 mg/kg, i.p.) was injected in order to facilitate recovery after the anaesthesia. After the surgery, rats were placed into a recovery chamber (Vet Tech Solutions Ltd) ventilated with oxygen. In coronary artery ligated (CAL) animals, approximately 92% survived and were able to meet the inclusion criteria for the experiments on isolated heart. All sham operated animals survived to 16 weeks after surgery. Animals were monitored and cared for daily. Sixteen weeks after surgery, the animals were used for experiments on isolated heart as shown in the Methods.

#### Experimental Groups

**Figure 1:**
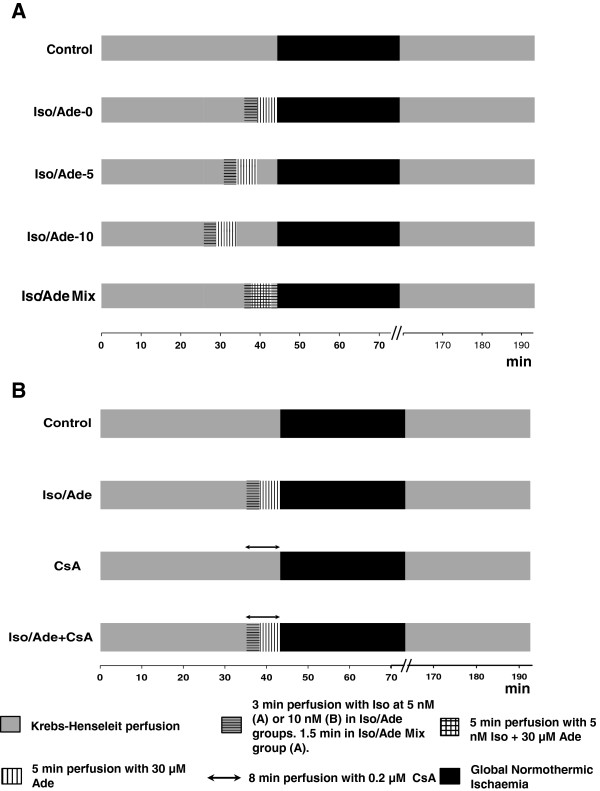
**Outline of the experimental protocols of series 1 and 2. Panel A** – experimental protocol of the Series 1. Iso/Ade-0, Iso/Ade-5 and Iso/Ade-10 – consecutive perfusion with isoproterenol and adenosine with 0, 5, and 10 min washout period respectively; Iso/Ade Mix – perfusion with the mixture of isoproterenol and adenosine. **Panel B** – experimental protocol of the Series 2. Control; Iso/Ade – consecutive isoproterenol/adenosine treatment with no washout period (similar to that of Iso/Ade-0 in the Series 1); CsA – perfusion with cyclosporine A; and Iso/Ade + CsA – consecutive isoproterenol/adenosine treatment combined with perfusion with Cyclosporine A.

**Figure 2:**
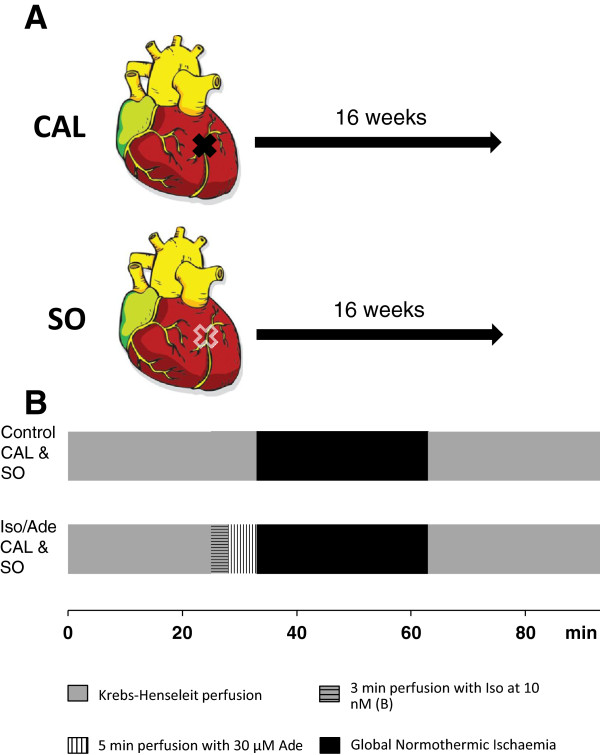
**Outline of the experimental protocol of series 3. Panel A** – model of surgically-induced heart failure. CAL – left anterior descending coronary artery ligation; SO – sham-operated heart. **Panel B** – experimental protocol of series 3. The CAL and SO hearts were divided to two groups each: Control CAL, Iso/Ade CAL, Control SO and Iso/Ade SO. The Iso/Ade protocol was similar to those of Iso/Ade-0 group in the Series 1 (Figure 1A) and Iso/Ade group in Series 2 (Figure 1B).

After a 25 min period of equilibration followed by preischaemic interventions, global normothermic ischaemia (37°C) was induced for 30 min and then normothermic perfusion was reinstated for 2 hr. Three series of experiments were performed as detailed below and summarised in Figures 1 and 2. In Series 1 and 2, experiments were carried out on healthy male Wistar rats (250–275 g) whilst in Series 3 rats with surgically-induced heart failure or sham-operated rats were employed.In Series 1 (Figure 1A), hearts were divided into a Control group and 4 treatment groups (8–10 hearts/group) according to the preischaemic protocol. Hearts of the Control group were not subjected to any intervention prior to ischaemia. Hearts of the three treatment groups, after the equilibration period, were subjected to the consecutive perfusion with Iso and Ade (3 min with 5 nM Iso and 5 min with 30 μM Ade) followed by a washout period of different duration: 10 min (Iso/Ade-10); 5 min (Iso/Ade-5) and zero min (Iso/Ade-0). Hearts of the fourth treatment group ((Iso/Ade Mix)) were perfused with Ade for 1.5 min and then 3 min with the mixture of Iso and Ade followed by another 1.5 min period of perfusion with Ade (Figure 1A). Analysis of haemodynamic function recovery, lactate dehydrogenase (LDH) release during reperfusion and infarct size, in this and the other series of experiments, was performed in all hearts subjected to ischaemia. Additional hearts (n = 9-10/group) of Control, Iso/Ade-10 and Iso/Ade-0 groups were freeze-clamped following 43 min preischaemia, ground under liquid nitrogen and stored at −80°C for later analysis of PKC activity. Another set of Control, Iso/Ade-0 and Iso/Ade Mix hearts (n = 7-8 in each group) were freeze-clamped at the end of the preishaemic protocol and used for the measurement of glycogen content.In Series 2 experiments, four groups (6–9 hearts/group) were employed (Figure 1B). Two of the groups were Control and Iso/Ade with no washout period. In this series of experiments and in the Series 3 experiments, Iso was used at 10 nM. In preliminary experiments (data not shown), we found that this concentration of Iso might give even higher cardioprotective effect than 5 nM, which is important in terms of optimisation of the intervention, yet was still in the range of normal pharmacological dose. Another two groups of hearts were perfused with 0.2 μM CsA for 8 min prior to ischaemia with or without the Iso/Ade treatment (Iso/Ade + CsA and CsA groups respectively).Series 3 experiments were carried out on CAL and SO rats. Hearts were divided into four groups (5–6 hearts/group): Control and Iso/Ade groups of CAL and SO rats. The experimental protocol of Control and Iso/Ade groups (Figure 2) in this series of experiments was similar to those in Series 2 (Figure 1B). Infarct size was not measured in these experiments because the CAL hearts had already developed an infarct as a result of the left descending coronary artery ligation.

Five additional CAL and 6 SO rats were used to confirm the development of heart failure. Body weight and tibia length were measured before the surgery and 16 weeks after the operation. After 16 weeks post-operation, the rats were killed by stunning and cervical dislocation, the hearts and lungs excised and the wet weight of the hearts, left ventricles and lungs were measured.

### Assays

#### PKC activity

PKC activity was determined in freeze-clamped heart powders using a kit supplied by Promega according to the manufacturer’s instructions. The assay relies on a change in charge of the fluorescent PepTag® C1 peptide from +1 to −1 following phosphorylation. Bands were visualized under UV light and the ratio of fluorescence intensity of phosphorylated to non-phosphorylated peptide was quantified using AlphaInotech ChemiImager 4400 with AlphaEase v5.5 software.

#### Glycogen content in myocardium

Glycogen content was assessed by measuring the glucose released from glycogen breakdown by amyloglucosidase based on the method described by Passonneau [16] and expressed as μmol glucose per gram tissue. A sample (70 mg) of the frozen tissue was suspended in 1 ml of 0.3 M perchloric acid (4°C), homogenized with a Polytron and 50 μl of the suspension were incubated with 0.5 ml of 50 mM Na Acetate Buffer (pH 5.5) containing 0.02% BSA and 50 μg/ml amyloglucosidase (Sigma) at room temperature for 2 hr. The samples were then centrifuged at 10000 *g* for 10 min and glucose content was assessed using a Glucose (HK) Assay Kit (Sigma) according to the manufacturer’s instruction. Briefly, 0.4 ml of the supernatant was mixed with 1 ml of the glucose assay reagent. Glucose content was measured spectrophotometrically (340 nm) after incubation at room temperature for 15 min.

The quantity of glucose released from glycogen breakdown was determined by subtracting glucose in samples incubated without amyloglucosidase from those incubated with the enzyme.

#### LDH activity

LDH activity was determined in the effluent perfusate collected from the hearts of all groups prior to ischemia and during each 5 min over the first 30 min of reperfusion as described earlier [17] and modified at our lab: 80 μl of the sample was added to 910 μl of the buffer (pH 7.4) containing 100 mM triethanolamine and 100 μM reduced β-nicotinamide adenine dinucleotide (NADH). After addition of 10 μl of 0.1 M sodium pyruvate to the reaction mixture, the change of absorbance at 340 nm (A_340_) was recorded using a spectrophotometer over 10 min at 37°C. LDH activity was calculated according to the rate of A_340_ decrease.

#### Infarct Size

Infarct size was determined as described previously [18]. Hearts were stained with 1% of triphenyltetrazolium chloride after 2 h of reperfusion, frozen at -20°C and sliced into 6 slices. Necrotic and intact areas of each side for each of heart slices were determined using AlphaEase v5.5 software and the total necrotic and intact area of ventricular myocardium of each heart was calculated. Since the entire heart was at risk from global ischemia, the infarct size was expressed dividing the sum of necrotic areas by the sum of total slice areas of the 6 slices to obtain the percentage of necrosis.

### Statistical Analysis

Data are presented as mean ± SEM. Statistical significances of the differences between groups were evaluated by one-way ANOVA followed by Tukey's multiple comparison post hoc test or two-tailed unpaired Student’s *t*-test (morphometric measurements of CAL and SO rats in the Series 3 experiments) using the software GraphPad Prism, Version 4.03. Differences were considered significant where P < 0.05.

## Results

### Optimization of the protocol of Iso/Ade treatment with a clinically relevant dose of Isoproterenol

**Figure 3:**
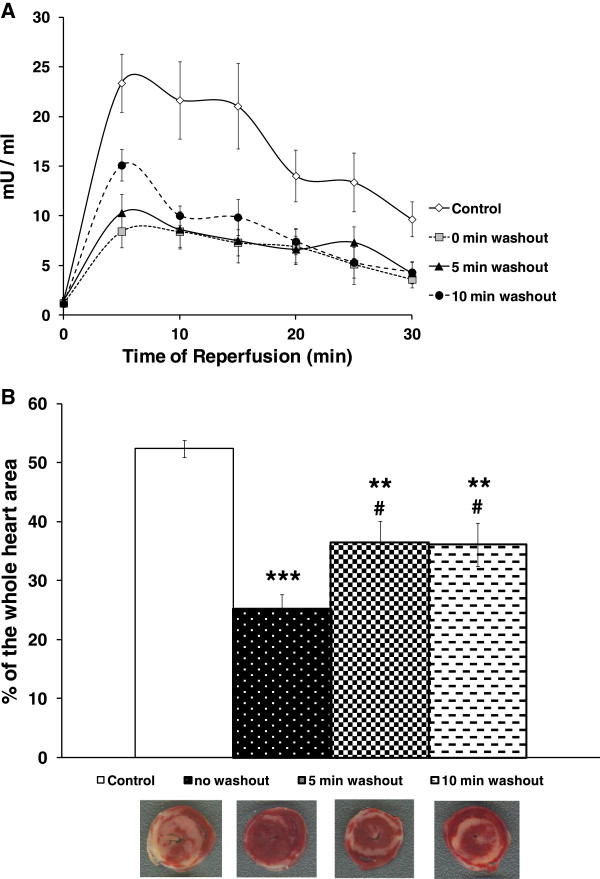
**Lactate dehydrogenase (LDH) activity and infarct size in hearts of the Series 1.** The experiments were carried out on Control (n = 7) and Iso/Ade groups with 0 (n = 9), 5 (n = 9), and 10 (n = 9) min washout periods. **Panel A** - LDH activity in the effluent perfusate during 30 min reperfusion. Differences between Control and all three Iso/Ade groups (measured for the corresponding data points) were statistically significant (P < 0.05) at all data points. There was no significant difference in LDH activity between the Iso/Ade groups. **Panel B** – Infarct size. Mean ± SEM (columns) and representative images of the corresponding heart slices stained with 2,3,5-triphenyltetrazolium chloride (TTC). ** P < 0.01, *** P < 0.001 *vs.* Control; # P < 0.05 *vs.* Iso/Ade group with zero min washout period.

**Figure 4:**
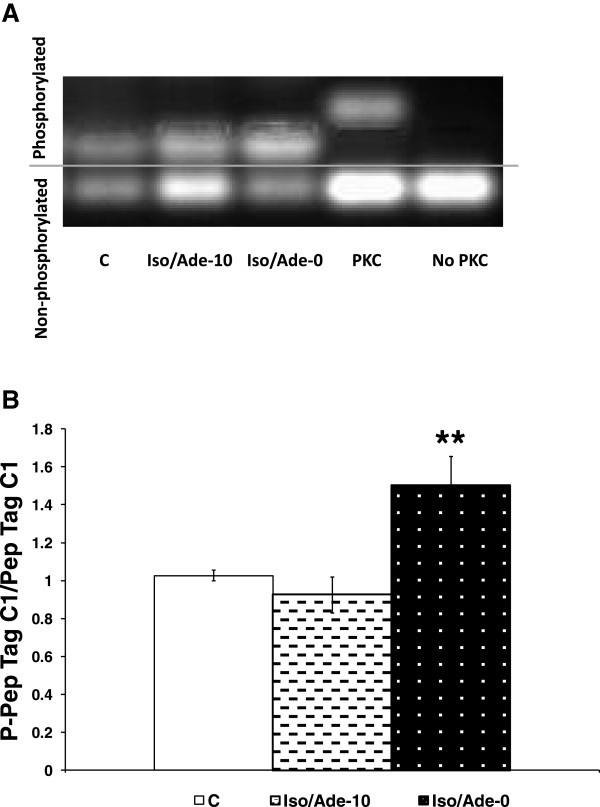
**Protein Kinase C (PKC) activity in hearts of Experimental Series 1.** PKC activity was measured using non-radioactive PepTag® assay and is expressed as Mean ± SEM of a ratio of fluorescence intensity of phosphorylated and non-phosphorylated PepTag® C1 peptide **(Panel B)** which is proportional to the PKC activity. PKC activity was measured in Control (C, n = 10), Iso/Ade-0 (n = 9) and Iso/Ade-10 (n = 9) groups. **Panel A** – Representative image of the phosphorylated and non-phosphorylated PepTag® C1 peptide under UV light. ** P < 0.01 *vs.* Control.

**Figure 5:**
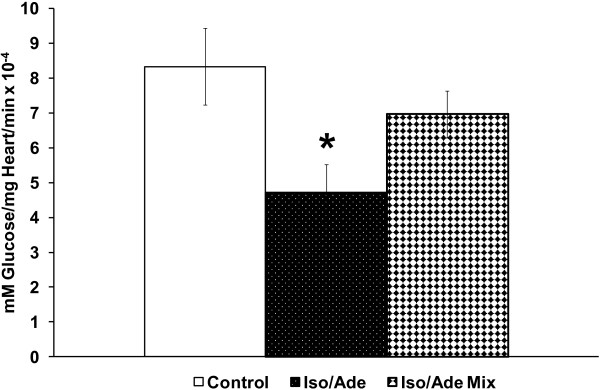
**Glycogen content in hearts of Experimental Series 1.** Glycogen content in myocardium of Control hearts (n = 8), hearts treated consecutively with Iso and Ade (Iso/Ade group, n = 8) and hearts treated with the mixture of Iso and Ade (Iso/Ade Mix, n=7) was measured prior to ischaemia. * P < 0.05 *vs.* control.

In the Iso/Ade group, RPP during heart perfusion with 5 nM Iso reached a maximum of 259.4 ± 12.4% of the pre-treatment value, mostly due to an increase in LVDP. RPP measured at the end of perfusion with 30 μM Ade fell to 42.3 ± 7.0% of the pre-treatment value, due to a significant reduction of both HR and LVDP. In Table 1, we show that during reperfusion, recovery of RPP in the Control group was 28%. However, perfusion with low Iso concentration (5 nM) followed by 30 μM Ade immediately prior to rendering the heart ischaemia (Iso/Ade-0 group), improved recovery in RPP to 76%. Similar recovery was achieved when the duration of perfusion prior to commencing ischaemia was extended to 5 or 10 min (Iso/Ade-5 and Iso/Ade-10 groups).The improved RPP recovery during reperfusion was accompanied by considerably reduced LVEDP and reflected improved recovery of LVDP with no significant change in HR. The time derivative during contraction was significantly higher in all three Iso/Ade groups than in Control group, whereas recovery of -dP/dt was significantly improved only in hearts of the Iso/Ade-0 group. The parameters of haemodynamic function recovery did not differ significantly between the three Iso/Ade groups but were the highest in Iso/Ade-0 group. Protection against necrotic damage showed a similar pattern to the recovery of haemodynamic function. Thus, LDH release during the first 30 min of reperfusion (Figure 3A) and infarct size at the end of reperfusion (Figure 3B) were significantly lower in Iso/Ade groups compared to Control. However infarct size in the group Iso/Ade-0 was notably lower compared to both Control and Iso/Ade-5 groups.PKC activity in Iso/Ade-0 group, measured at the end of the pre-ischaemic protocol, was 1.5 fold higher compared to both Control and Iso/Ade-10 groups (Figure 4).Glycogen content was halved in hearts treated consecutively with Iso and Ade, but simultaneous perfusion of hearts with these drugs had no significant effect on this parameter (Figure 5).

**Table 1:** Haemodynamic function in Iso/Ade groups of hearts with different duration of the washout period

**Parameters**	**Preiscaemic equilibration (n = 34)**	**Reperfusion (Percentage preischaemic value)**				
		**Control (n = 7)**		**Iso/Ade-0 (n = 10)**	**Iso/Ade-5 (n = 9)**	**Iso/Ade-10 (n = 8)**
LVDP (mmHg)	75.1 ± 2.5	% of initial values	29.4 ± 5.7	88.9 ± 7.2 ***	73.6 ± 8.3 *	78.3 ± 14.9 *
HR (beat/min)	277.6 ± 4.2		102.5 ± 11.4	86.9 ± 5.6	94.5 ± 3.2	71.6 ± 8.9
RPP (mmHg · beat/min)	20802 ± 713		28.5 ± 5.2	76.3 ± 6.9 ***	68.5 ± 7.0 **	73.3 ± 10.5 **
+dP/dt (mmHg/s)	3082 ± 117		30.2 ± 2.5	65.2 ± 6.9 **	58.6 ± 5.3 *	60.4 ± 8.5 *
-dP/dt (mmHg/s)	−2027 ± 83		42.7 ± 4.4	85.5 ± 7.2 **	65.6 ± 8.2	71.5 ± 8.9
Maximal LVEDP at Reperfusion (mmHg)	-	98.7 ± 7.5		50.0 ± 4.4 ***	62.1 ± 8.2 **	63.2 ± 6.5 **

Haemodynamic function was determined at the end of the equilibration period prior to ischaemia and after 60 min reperfusion.

* P < 0.05, ** P < 0.01, *** P < 0.001 *vs.* Control.

### Effect of the combined treatment with Iso, Ade and cyclosporine A on ischaemia/reperfusion injury

**Figure 6:**
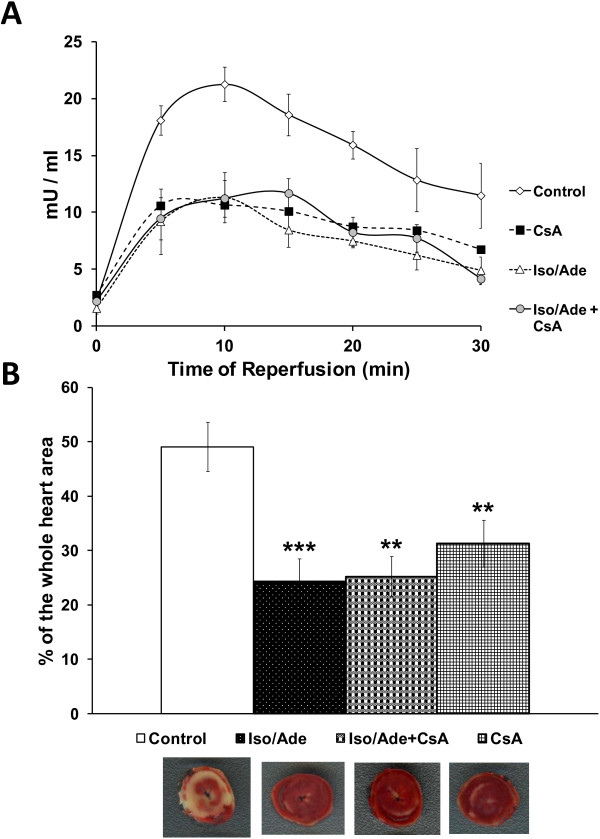
**LDH activity and infarct size in hearts in Experimental Series 2.** The experiments were carried out on Control (n = 9), Iso/Ade (n = 9), cyclosporine A (CsA, n = 6) and Iso/Ade + CsA (n = 8) groups of hearts. The perfusion protocol of Iso/Ade group in this series of experiments was similar to that of Iso/Ade-0 group in Experimental Series 1. **Panel A** – LDH activity in the effluent perfusate during 30 min reperfusion. Differences between control and three other groups (measured for the corresponding data points) were statistically significant (P < 0.05) at all data points. There was no significant difference in LDH activity between the three intervention groups. **Panel B** – Infarct size. Mean ± SEM (columns) and representative images of the corresponding heart slices stained with TTC. ** P < 0.01, *** P < 0.001 *vs.* Control.

In the Iso/Ade group of the second series of experiments, RPP during heart perfusion with 10 nM of Iso reached a maximum of 346.1 ± 26.9% of the pre-treatment value, significantly exceeding RPP increase in Series 1 hearts treated with 5 nM Iso (P < 0.05). This elevation of RPP was mostly due to increase in LVDP. RPP measured at the end of perfusion with 30 μM Ade fell to 36.8 ± 7.6% of the pre-treatment value due to a significant reduction of both HR and LVDP. The MPTP inhibitor CsA significantly increased RPP recovery compared to Control (Table 2) and was as effective as the consecutive Iso/Ade treatment in terms of reduction of infarct size (Figure 6B). LDH activity in the effluent perfusate during reperfusion was also significantly reduced in these hearts (Figure 6A). However perfusion of hearts with CsA together with the consecutive Iso/Ade treatment (Iso/Ade + CsA group), at clinically relevant concentration of these drugs, did not bring about any additive protective effect; infarct size and LDH release were similar to those of Iso/Ade group.

**Table 2:** Haemodynamic function in hearts of Control and Iso/Ade groups with or without cyclosporine A

**Parameters**	**Pre-Ischaemia (n = 24)**	**Reperfusion (Percentage preischaemic value)**				
		**Control (n = 7)**		**Iso/Ade (n = 6)**	**CsA (n = 5)**	**Iso/Ade + CsA (n = 6)**
LVDP (mmHg)	75.8 ± 2.9	% of initial values	39.0 ± 6.6	100.5 ± 14.2 *	89.5 ± 14.4	97.8 ± 16.9 *
HR (beat/min)	288.3 ± 3.9		96.0 ± 3.5	93.1 ± 3.1	99.3 ± 4.7	102.9 ± 2.9
RPP (mmHg · beat/min)	21854 ± 889		38.8 ± 7.5	93.0 ± 12.0 **	86.3 ± 13.3 *	100.3 ± 11.2 **
+dP/dt (mmHg/s)	2969 ± 94		34.2 ± 4.6	81.3 ± 12.3 *	66.4 ± 13.3	87.2 ± 14.3 *
-dP/dt (mmHg/s)	−2074 ± 84		42.7 ± 4.7	93.6 ± 12.4 **	71.7 ± 8.3	90.8 ± 13.5 *
Maximal LVEDP at Reperfusion (mmHg)	-	98.6 ± 5.6		53.9 ± 13.2 **	78.2 ± 8.6	65.9 ± 4.2 *

Haemodynamic function was determined at the end of equilibration period prior to ischaemia and after 60 min reperfusion. The perfusion protocol of Iso/Ade group in this series of experiments was similar to that of Iso/Ade-0 group in Experimental Series 1.

* P < 0.05, ** P < 0.01, *** P < 0.001 *vs.* Control.

### Consecutive Iso/Ade treatment protects hearts with surgically-induced heart failure

At the time of the ligation of left coronary artery, no significant difference in body weight or tibia length was detected between sham-operated (SO) and Coronary Artery Ligation (CAL) animals. After 16 weeks post-surgery, there was no difference in body weight between the two groups. However, significant increases in heart and lung weights, heart and lung to body weight ratios, as well as tibia length to body weight ratio in the CAL animals were observed (Table 3) indicating both cardiac hypertrophy and cardiac insufficiency.

**Table 3:** Effects of the left coronary artery ligation (CAL) on cardiac morphology

**Procedure**	**BW (g)**	**HW (g)**	**LW (g)**	**Tibia (mm)**	**HW/BW (mg/g)**	**LW/BW (mg/g)**	**HW/Tibia (mg/mm)**	**LW/Tibia (mg/mm)**
Sham (n = 6)	502.2 ± 8.0	1.56 ± 0.04	1.62 ± 0.04	46.7 ± 0.5	3.1 ± 0.1	3.2 ± 0.1	3.3 ± 0.1	3.5 ± 0.1
CAL (n = 5)	491.4 ± 25.6	2.42 ± 0.20 **	1.97 ± 0.13 *	46.1 ± 0.6	4.9 ± 0.4 ***	4.0 ± 0.1 ***	5.3 ± 0.4 ***	4.3 ± 0.2 **

BW – body weight; HW – heart weight; LW – lung weight. * P < 0.05, ** P < 0.01, *** P < 0.001 *vs.* Sham.

**Figure 7:**
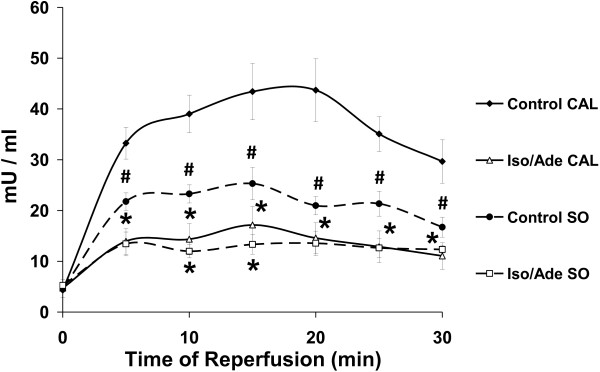
**LDH activity in hearts of Experimental Series 3.** LDH activity was measured in the effluent perfusate during 30 min reperfusion. The perfusion protocol of Iso/Ade groups in this series of experiments was similar to that of Iso/Ade-0 group in the Experimental Series 1. LDH activity is presented for Control and Iso/Ade groups in sham-operated hearts (SO, n = 6 in each group) and failing hearts after the left descending coronary artery ligation (CAL, n = 5 in control and n = 6 in Iso/Ade group). * P < 0.05 *vs.* corresponding Control (either SO or CAL); # P < 0.05 *vs.* Control CAL.

During heart perfusion with 10 nM of Iso, RPP reached a maximum of 401.3 ± 30.8% of the pre-treatment value in SO group and 343.3 ± 52.4% in CAL group, mostly due to increase in LVDP. This parameter in the SO group, but not in the CAL group, was significantly higher than in Series 1 where the hearts were treated with 5 nM Iso (P < 0.01). RPP measured at the end of perfusion with 30 μM Ade fell to 43.5 ± 8.0% of the pre-treatment value in SO hearts and to 56.6 ± 9.3% in CAL hearts, due to a significant reduction of HR and return of LVDP to the pre-treatment value. There was no significant difference in changes of RPP in response to the perfusion with Ade between SO and CAL groups. Haemodynamic function recovery of both SO and CAL groups of hearts following ex-vivo ischaemia/reperfusion was very poor and did not significantly differ from each other (Table 4). Thus, LVDP and RPP reached only 20–25% of the initial preischaemic values. Iso/Ade treatment significantly improved haemodynamic function recovery of both SO and CAL hearts with RPP reaching 70% of the preischaemic value. LDH activity during reperfusion was highest in Control CAL hearts and was significantly higher not only than Iso/Ade treated hearts of SO and CAL groups but also than Control SO hearts (Figure 7). Thus, necrotic damage was greatest in the untreated failing hearts but Iso/Ade treatment was able to protect the failing hearts at least as effectively as the sham-operated ones.

**Table 4:** Haemodynamic function in coronary artery ligation (CAL) and sham-operated (SO) Iso/Ade groups of hearts

**Parameters**	**Pre-Ischaemia (n = 20)**	**Reperfusion (Percentage preischaemic value)**				
		**Control SO (n = 5)**		**Iso/Ade SO (n = 5)**	**Control CAL (n = 5)**	**Iso/Ade CAL (n = 5)**
LVDP (mmHg)	75.2 ± 4.3	% of initial values	24.1 ± 3.9	65.4 ± 9.1 *	20.7 ± 4.0	69.6 ± 15.2 **
HR (beat/min)	271.1 ± 7.8		89.1 ± 19.4	107.5 ± 7.6	94.4 ± 3.0	99.0 ± 4.5
RPP (mmHg · beat/min)	20454 ± 1333		22.5 ± 5.9	66.3 ± 9.8 *	19.7 ± 4.0	69.0 ± 16.3 *
+dP/dt (mmHg/s)	2596 ± 154		31.7 ± 3.3	82.4 ± 20.8	66.3 ± 15.8	87.2 ± 14.3
-dP/dt (mmHg/s)	−1868 ± 138		37.8 ± 2.7	86.5 ± 22.1 **	40.4 ± 4.5	77.0 ± 14.3
Maximal LVEDP at Reperfusion (mmHg)	-	91.9 ± 19.1		28.7 ± 12.2 *	67.3 ± 9.9	20.2 ± 21.1

Haemodynamic function was determined at the end of equilibration period prior to ischaemia and after 60 min reperfusion. The perfusion protocol of Iso/Ade groups in this series of experiments was similar to that of Iso/Ade-0 group in Experimental Series 1.

* P < 0.05, ** P < 0.01 *vs.* a corresponding Control.

## Discussion

### Optimisation of the Iso/Ade treatment and its translational significance

Our results show that Iso at its low dose (5–10 nM), followed by the perfusion with 30 μM Ade, can successfully protect hearts against ischaemia/reperfusion injury. In fact, when Iso was perfused at 10 nM, the protective effect was similar to the higher concentration of Iso (200 nM) used in our previous experiments [4]. Since both Iso and Ade are used in clinical settings and can protect hearts at clinically relevant doses, the consecutive Iso/Ade treatment has strong potential for translation into clinical practice. Furthermore, the use of these drugs at low doses will minimise other drug interactions. This work also demonstrates that there is a window of at least 10 min within which the index ischaemia can be started and strong cardioprotection still be observed. However, it is clear that, the shorter the period between the end of the treatment and the start of ischaemia the higher the cardioprotective efficacy of the treatment will be. Thus in our experiments, infarct size in hearts with no washout period (Iso/Ade-0 group) was significantly smaller than in both Control and Iso/Ade-5 hearts. Furthermore, only in Iso/Ade-0 hearts was -dP/dt recovery significantly higher than in controls. This might be explained by the index ischaemia in these hearts commencing while PKC remained activated, potentially leading to more effective survival signalling.

Having established that clinically-relevant doses of isoproteronol and adenosine are cardioprotective and was optimal when applied immediately prior to ischaemia, the next step was to identify the target of this powerful cardioprotective intervention. Our earlier work suggested that reduced ROS production and consequent inhibition of MPTP opening were likely targets [4]. If this is the case, protection should not be further enhanced by direct inhibition of the MPTP by Cyclosporin A and we confirmed this to be the case.

### Role of MPTP and Cardiac Glycogen in cardioprotection by Iso/Ade treatment

We [9] and others [19-21] have shown that MPTP opening is a key factor of irreversible damage during reperfusion and that its prevention underlies cardioprotection by a variety of preconditioning [12] and post-conditioning protocols [10]. Thus, it was hypothesized that the protection associated with ischaemic preconditioning could be attributed to a decreased probability of MPTP opening [9,22]. Ovize et al. proposed MPTP inhibition as the effector of ischaemic postconditioning [10]. It has been shown that the protective effect of remote ischaemic preconditioning [11] and a variety of pharmacologically-induced cardioprotective interventions [12] also rely on the MPTP inhibition. In our recent study with Iso/Ade treatment, where Iso was used at relatively high concentration (200 nM), the treatment resulted in a dramatic reduction of calcium-induced mitochondria swelling implicating MPTP inhibition as an end effector of the cardioprotective mechanism [4].

In the present work, we used the MPTP inhibitor CsA in order to confirm a role for MPTP inhibition in the cardioprotection afforded by Iso/Ade treatment. We found that CsA improved RPP recovery and significantly reduced necrotic damage to myocardium during reperfusion. These results are consistent with earlier data shown by us [23,24] and others [25-27] confirming a cardioprotective effect of CsA. However, no additive cardioprotective effect was seen when consecutive treatment with 10 nM isoproterenol and 30 μM adenosine was combined with CsA treatment. These data suggest that cardioprotection by the Iso/Ade treatment is mediated through inhibition of the MPTP.

It has been reported that protection via ischaemic preconditioning is associated with glycogen depletion leading to less anaerobic glycolysis during the subsequent prolonged ischaemia, and hence reduced accumulation of lactate and H^+^. This causes a smaller decrease in intracellular pH during ischaemia and thus less compensatory increases in intracellular Na^+^ and Ca^2+^[28]. The decreased Ca^2+^ loading will reduce the likelihood that the MPTP opens which might partially explain the observed cardioprotection [29]. Glycogen breakdown has also been linked to attenuation of the loss of hexokinase 2 (HK-2) binding to mitochondria that occurs during ischaemia [30]. The binding of HK-2 plays an anti-apoptotic role and inhibits MPTP opening [31,32] and there is an inverse correlation between the extent of HK-2 binding at the end of ischaemia and infarct size [30]. Increase HK-2 binding at the end of ischaemia can be mediated, at least in part, by decreasing tissue glycogen content prior to ischaemia. This leads to a reduction in tissue glucose-6-phosphate levels and acidification during ischaemia, which mediate HK-2 dissociation from mitochondria [30]. The data of Figure 5 confirm that the consecutive Iso/Ade treatment also depleted glycogen content considerably consistent with this mechanism, whereas the reduction in glycogen content following simultaneous application of the drugs was not significant and was accompanied by considerably less cardioprotection. However, reduced glycogen content cannot entirely account for the cardioprotection since we have shown previously that a similar decrease in glycogen could be induced by high dose of isoproterenol alone, yet this treatment was less cardioprotective than the consecutive treatment with isoproterenol and adenosine [4]. Thus adenosine may contribute to cardioprotection through glycogen independent mechanisms that improve haemodynamic function recovery during reperfusion and inhibit MPTP opening [4].

### Consecutive Iso/Ade treatment protects hearts with surgically-induced heart failure

Since the majority of patients undergoing cardiac surgery suffer from co-morbid illnesses, for clinical use of consecutive Iso/Ade treatment it is necessary to confirm its efficacy in an experimental model of the diseased heart [33,34]. In general, the presence of co-morbid illness renders the myocardium resistant to cardioprotection against infarction by physical or pharmacological stimuli [14]. It has been found that compensated left ventricular hypertrophy does not compromise the efficacy of cardioprotective interventions such as ischaemic preconditioning but heart failure abolishes any beneficial effect [33,35,36]. Heart failure is a complex clinical syndrome, the etiology of which generally stems from pre-existing ischaemic heart/coronary artery disease, or it is of non-ischaemic and/or idiopathic origin [37,38]. It is characterized by the progressive inability of the heart to fill with, and eject adequate amounts of blood to meet the needs of the body [39].

Ligation of the left descending coronary artery in our experiments rendered a significant increase in heart and lung weight to body weight and tibia length ratios (Table 3) in 16 weeks after the surgery indicating both cardiac hypertrophy and cardiac insufficiency.Consistent with the dose-dependent effect, preischaemic perfusion of SO hearts with 10 nM Iso resulted in a significantly higher increase of RPP than in hearts of Series 1 treated with 5 nM Iso. In the CAL group, RPP was also increased considerably during perfusion with 10 nM Iso but this was not significantly higher than in hearts treated with 5nM Iso. Thus, although the myocardium of rats with surgically-induced heart failure model showed somewhat reduced β-adrenergic reactivity, they still responded well to the β-adrenergic stimulation. Control CAL hearts suffered more severe necrotic damage during reperfusion than their sham-operated counterparts as reflected in the released LDH activity during reperfusion which was twice as high in these hearts as compared to the non-treated SO hearts (Figure 7). Nevertheless, the haemodynamic function recovery in Iso/Ade treated CAL hearts was similar to SO hearts whilst LDH release was suppressed more strongly than in the sham-operated hearts. This implies that heart failure in our experiments did not compromise the cardioprotective effect of consecutive Iso/Ade treatment and that the survival signal transduction pathways activated by this treatment were still responsive in spite of the developed heart failure.

The results of our study may represent a significant step towards translation of the consecutive Iso/Ade treatment to cardiac surgery involving ischaemic cardioplegic arrest and cardiopulmonary bypass.

## Conclusions

The novel findings of our study are as follows:

1. Strong cardioprotective effect of PKA/PKC activation can be achieved by heart perfusion with Iso at the clinically relevant concentration of 5–10 nM followed by perfusion with Ade.

2. The cardioprotective efficacy of PKA/PKC activation remains for at least 10 min after the intervention. From a practical point of view, this means that this protective intervention will still be effective even if it is performed some time before the ischaemic incident (*i.e.* cardiac surgery with cardioplegic arrest).

3. In contrast to a number of studies showing loss or attenuation of cardioprotection in diseased or ageing hearts, consecutive Iso/Ado treatment can protect the failing heart effectively. This efficacy of the treatment in a relevant comorbidity model increases the likelihood of successful translation into clinical practice.

4. Our data suggest a key role of MPTP inhibition in the cardioprotective effect of this treatment. Glycogen depletion of the myocardium induced by the treatment prior to ischaemia is implicated in the mechanism underlying MPTP inhibition and cardioprotection.

## Abbreviations

Ade: Adenosine; CAL: Coronary artery ligated; CsA: Cyclosporine A; Iso: Isoproterenol; KH: Krebs-Henseleit buffer; LDH: Lactate dehydrogenase; LVDP: Left ventricular developed pressure; LVEDP: Left ventricular end-diastolic pressure; LVSP: Left ventricular systolic pressure; MPTP: Mitochondria permeability transition pore; PKA: Protein kinase A; PKC: Protein kinase C; SO: Sham-operated; RPP: Rate-pressure product; TP: Temperature preconditioning; TTC: 2,3,5-triphenyltetrazolium chloride; +dP/dt: Time derivatives of pressure during contraction; -dP/dt: Time derivative of pressure during relaxation.

## Competing interests

The authors declare that they have no competing interests.

## Authors’ contributions

IK participated in design of the study, carried out data acquisition, data analysis and drafted the manuscript. APH participated in design of the study and helped to draft the manuscript. SMB provided the surgically-induced model of heart failure. DJD took part in the data acquisition and data analysis. AFJ participated in design and coordination of the study and helped to draft the manuscript. MSS conceived the study, participated in its design and coordination and helped to draft the manuscript. All authors have read and approved the final manuscript.
